# *Edwardsiella tarda* OmpA Encapsulated in Chitosan Nanoparticles Shows Superior Protection over Inactivated Whole Cell Vaccine in Orally Vaccinated Fringed-Lipped Peninsula Carp (*Labeo fimbriatus*)

**DOI:** 10.3390/vaccines4040040

**Published:** 2016-11-07

**Authors:** Saurabh Dubey, Kiran Avadhani, Srinivas Mutalik, Sangeetha Madambithara Sivadasan, Biswajit Maiti, Shivani Kallappa Girisha, Moleyur Nagarajappa Venugopal, Stephen Mutoloki, Øystein Evensen, Indrani Karunasagar, Hetron Mweemba Munang′andu

**Affiliations:** 1 Department of Fisheries Microbiology, Karnataka Veterinary, Animal & Fisheries Sciences University, College of Fisheries, Mangalore 575002, India; saurabh.dubey@nmbu.no (S.D.); sangeethasivadas888@gmail.com (S.M.S.); skgirisha@gmail.com (S.K.G.); mnvenu@rediffmail.com (M.N.V.); 2 Norwegian University of Life Sciences, Faculty of Veterinary Medicine, Department of Basic Sciences and Aquatic Medicine, Section of Aquatic Medicine and Nutrition, Adamstuen Campus, Ullevålseveien 72, P.O. Box 8146, NO-0033 Dep, Oslo 0454, Norway; stephen.mutoloki@nmbu.no (S.M.); oystein.evensen@nmbu.no (Ø.E.); 3 Department of Pharmaceutics, Manipal College of Pharmaceutical Sciences, Manipal University, Manipal 576104, Karnataka State, India; kirankle84@gmail.com (K.A.); ssmutalik@yahoo.com (S.M.); 4 UNESCO MIRCEN for Marine Biotechnology, Nitte University Centre for Science Education and Research, Paneer Campus, Deralakatte, Mangalore 575018, India; maiti.b@nitte.edu.in (B.M.); indrani.karunasagar@nitte.edu.in (I.K.)

**Keywords:** carp, chitosan, nanoparticle, oral, outer membrane protein, vaccination

## Abstract

The use of oral vaccination in finfish has lagged behind injectable vaccines for a long time as oral vaccines fall short of injection vaccines in conferring protective immunity. Biodegradable polymeric nanoparticles (NPs) have shown potential to serve as antigen delivery systems for oral vaccines. In this study the recombinant outer membrane protein A (rOmpA) of *Edwardsiella tarda* was encapsulated in chitosan NPs (NP-rOmpA) and used for oral vaccination of *Labeo fimbriatus*. The rOmpA purity was 85%, nanodiameter <500 nm, encapsulation efficiency 60.6%, zeta potential +19.05 mV, and there was an in vitro release of 49% of encapsulated antigen within 48 h post incubation in phosphate-buffered saline. Empty NPs and a non-formulated, inactivated whole cell *E. tarda* (IWC-ET) vaccine were used as controls. Post-vaccination antibody levels were significantly (*p* = 0.0458) higher in the NP-rOmpA vaccinated fish (Mean OD_450_ = 2.430) than in fish vaccinated with inactivated whole cell *E. tarda* (IWC-ET) vaccine (Mean OD_450_ = 1.735), which corresponded with post-challenge survival proportions (PCSP) of 73.3% and 48.28% for the NP-rOmpA and IWC-ET groups, respectively. Serum samples from NP-rOmpA-vaccinated fish had a higher inhibition rate for *E. tarda* growth on tryptic soy agar (TSA) than the IWC-ET group. There was no significant difference (*p* = 0.989) in PCSPs between fish vaccinated with empty NPs and the unvaccinated control fish, while serum from both groups showed no detectable antibodies against *E. tarda*. Overall, these data show that the NP-rOmpA vaccine produced higher antibody levels and had superior protection over the IWC-ET vaccine, showing that encapsulating OmpA in chitosan NPs confer improved protection against *E. tarda* mortality in *L. fimbriatus*. There is a need to elucidate the possible adjuvant effects of chitosan NPs and the immunological mechanisms of protective immunity induced by OMPs administered orally to fish.

## 1. Introduction

*Edwardsiella tarda* is a member of the *Enterobacteriaceae* family that infects different fish species and mammals. In Channel catfish (*Ictalurus punctatus*), eels (*Anguilla anguilla*), and Japanese flounder (*Paralichthys olivaceus*), it causes emphysematous putrefaction, gangrene, and red disease [1]. It has been isolated from Red sea bream (*Pagellus bogaraveo*), Yellowtail (*Seriola lalandi*), Turbot (*Scophthalmus maximus*), Tilapia (*Oreochromis niloticus*), Mullet (*Mullus barbatus*), eels, Channel catfish, and Japanese flounder [1,2]. It is a zoonotic agent causing gastrointestinal and extra-intestinal infection in humans [3,4]. There are 61 different serovars of *E. tarda* identified based on somatic (O) and flagellar (H) antigens, infecting a wide range of hosts from different parts of the world [5]. In fish, there are no commercial vaccines available and, hence, there is a need for a conserved universal antigen for use in vaccine design. Bacterial outer membrane proteins (OMPs) are highly immunogenic and recognized as pathogen-associated molecular patterns (PAMPs) by pattern recognition receptors (PRRs) on host cells. They are conserved among different serovars [6,7] and have attracted a lot of interest in vaccine design. They serve as antigenic sites because of their exposed epitopes on the outer surfaces of bacterial cell membranes [8,9,10,11] and are suitable molecules for genetic engineering since they are made of simple structures that can be produced in inclusion bodies and easily recovered in the exact native conformation (12).

Although injectable inactivated bacterial vaccines bring about a significant decrease in disease outbreaks in aquaculture [12], the use of oral vaccines, which would be more practical, has been hampered by a general lack of efficacy [13]. Adjuvants have the advantage of enhancing the immunogenicity of non-replicative antigens; by reducing the quantity of antigens required per dose and forming depots at injection sites, they reduce the number of boosters required to induce long-term protective immunity [12,14]. Moreover, current advances in fish immunology show that the fish gut is endowed with antigen-presenting cells (APCs) and processing mechanisms comparable to those seen in lymphoid organisms [15,16,17]. However, the challenge in the design of oral vaccines for finfish has been developing formulations that protect the antigens from the harsh environment of the stomach and/or the foregut, thereby facilitating antigen uptake in the hindgut. An alternative that has attracted a lot of interest in recent years is the use of biodegradable polymeric nanoparticles that permit a sustained or pulsed release of encapsulated antigens. Among the polymers used in vaccine delivery are Poly(d,l-lactic-co-glycolic) acid (PLGA) [18,19] and chitosan [20,21]. Chitosan is a natural biodegradable polysaccharide obtained from crustacean shells and has been used for targeted drug [22] and DNA vaccine delivery [23,24,25]. In the present study an oral vaccine based on the recombinant OmpA (rOmpA) antigen was encapsulated in chitosan nanoparticles and tested for protective ability against *E. tarda* infection in *L. fimbriatus*. Hence, we wanted to determine whether oral vaccination using the rOmpA antigen encapsulated in chitosan nanoparticles would afford higher protection than the levels obtained in our previous studies, in which the rOmpA protein was intraperitoneally injected in fish without nanoparticle encapsulation. We used *L. fimbriatus* not only because it is a food fish but also because of its importance as an endangered species on the International Union of Conservation for Nature (IUCN) red list of threatened fish species [26]. The wild population of *L. fimbriatus* has significantly declined, becoming nearly extinct in areas of its original distribution due to overharvesting and river pollution. In order to prevent its further decline, current efforts are aimed at rearing *L. fimbriatus* in aquaculture, but these are hampered by disease outbreaks due to infectious agents such as *E. tarda*. Hence, there is a need to develop highly protective vaccines with the capacity to induce long-term protection in vaccinated fish in order to increase the population of this species for food and nutritional security.

## 2. Materials and Methods

### 2.1. Expression and Purification of OmpA of E. tarda

A recombinant OmpA (rOmpA) cloned in our lab at the UNESCO MIRCEN for Marine Biotechnology, Mangalore was expressed in *Escherichia coli* M15 cells [11]. The *E. tarda* isolate (Strain PCF01, GeneBank Acc. No. FJ751236.2) used for amplification of the rOmpA gene in the present study was obtained from catfish (*Pangasius hypohthalmus*) from east coast of India [11]. Briefly, characterization of the rOmpA was carried out by initially extracting the genomic DNA from the bacteria. Extraction of the OMP was carried out as previously described by Filip et al. [27] and the steps of amplification, cloning and expression followed by characterization has been detailed in our previous study [11]. Briefly, PCR amplification of the extracted OmpA was carried out using three different primers based on the OmpA of *E. tarda* strain CK41 (GenBank Acc. EF528483). The PCR reaction was done in a thermal cycler (Applied Biosystems, Carlsbad, CA, USA) using a master mix consisting of 5 μL of 10× buffer (100 mM Tris-HCl pH 8.3, 20 mM MgCl_2_, 500 mM KCl, 0.1% gelatin), 50 μM deoxynucleotide triphosphates (dNTPs), 2 U Taq polymerase, and 20 pmol of each primer. PCR conditions included an initial denaturation at 95 °C for 5 min followed by 35 cycles of denaturation for 1 min at 95 °C, annealing for 1 min at 60 °C , extension for 1 min at 72 °C , and a final delay at 72 °C for 5 min. PCR products were analyzed by gel electrophoresis using ethidium bromide and amplicons were purified for sequencing using the Qiagen kit (Qiagen, Hilden, Germany) according to the manufacturer’s guidelines. Sequence alignments for the amplicons purified in this study with strain CK41 used in the primer design were used to determine the sequence similarity of the gene product generated in this study.

Cloning of the OmpA gene was carried out by excluding the region coding for the signal peptide using PCR conditions described above, except for the annealing temperature, which was set at 51 °C. The purified products were ligated into the 30-UA commercial vector (Qiagen) set at 16 °C for 2 h. Thereafter, the plasmids were transformed into the competent M15 *E. coli* cells followed by heat shock and were later grown in LB broth containing kanamycin (100 μg/mL) and ampicillin (25 μg/mL). The bacteria cultures were induced by 1 mM isopropyl thiogalactoside (IPTG) and were grown until the turbidity reached 0.5–0.7 OD_600_. Analysis of recombinant expression of the OmpA protein was carried out using 12% SDS-PAGE as previously described [28]. For purification, IPTG induced recombinant cells were disrupted using the lysis buffer (6 M GuHCl; 0.1 M NaH_2_PO_4_; 0.01 M Tris-Cl; pH 8.0) while the cell debris was separated by centrifugation at 10,000× *g* for 30 min. The supernatant was mixed with 50% Ni–NTA slurry at a ratio of 4:1and added to columns. Purification was achieved by washing using a wash buffer (8 M urea; 0.1 M NaH_2_PO_4_; 0.01 Tris-Cl; pH 6.3 and 5.9) followed by eluting the purified protein using an elution buffer (8 M urea; 0.1 M NaH_2_PO_4_; 0.01 M Tris-Cl; pH 4.5). The purity of the protein was analyzed using 12% SDS-PAGE while the concentration was determined using the method previously described by Lowry et al. [28]. The immunogenicity of the cloned rOmpA was tested using a polyclonal antibody produced from immunized rabbits in our previous study [11].

### 2.2. Preparation of Chitosan Nanoparticles

Chitosan nanoparticles were prepared by an ionic gelation process, as described by Gan et al. [23], with minor modifications. Briefly, purified low molecular weight (75%–85% deacetylated) chitosan (Sigma-Aldrich^®^, St. Louis, MO, USA), was put in 1% (w/v) acetic acid to obtain a 0.15% (w/v) chitosan concentration. Thereafter, 10 mg rOmpA was put in the chitosan solution, adjusted to pH 5.5, and maintained at 20 ± 2 °C up to the time of making the chitosan nanoparticles. The chitosan-rOmpA solution was flush mixed with sodium TPP (sodium tripolyphosphate, Sigma-Aldrich^®^). The nanoparticles were formed spontaneously via the TPP initiated ionic crosslink and coacervation mechanism at a chitosan:TPP weight ratio of 3:1. Nanoparticles (NPs) encapsulated with rOmpA were separated by centrifuging at 14,000 rpm for 30 min at 14 °C, freeze-dried, and stored at 4 °C [23].

### 2.3. Characterization and Encapsulation Efficiency of Chitosan Nanoparticles

Chitosan particle size was determined using the Malvern ZetaSizer (NanoZS, Malvern Instruments, Worcester, UK) particle estimator while the binding capacity of rOmpA was determined by completely dissolving the nanoparticle in 0.1 M NaOH containing 0.5% w/v SDS. After lyophilization, the size and zeta potential of chitosan NPs was measured using a zeta analyzer. One milliliter of the supernatant obtained during chitosan preparation was kept to check the loading efficiency. Released antigen was quantified using the method of Lowry et al. [28]. The protein encapsulation efficiency was determined using the following equation: Encapsulation Efficiency = [(Total amount of Protein − free amount Protein in supernatant)/total amount Protein] × 100.

### 2.4. In Vitro Release Test

The in vitro release test was performed to determine the timing of antigen release from the chitosan nanoparticles according to the method of Hori et al. [29] with minor modifications. Briefly, 10 mg of chitosan nanoparticles encapsulating rOmpA were suspended in 500 μL phosphate buffer saline (PBS). The vial was shaken horizontally in a water bath at 37 °C for up to 48 h. In vitro protein release was determined by drawing 500 μL supernatant, which was replaced with an equal volume of PBS, after the centrifugation at 10,000× g for 10 min. Samples were collected after 1, 2, 8, 16, 24, and 48 h. Released antigen was quantified using the method of Lowry et al. [28].

### 2.5. Vaccine Preparation for Oral Immunization

The chitosan NPs oral vaccine was prepared by mixing the rOmpA antigen with commercial feed for carp by powdering the feed using a grinder followed by sieving. The vaccine–feed mixture was thoroughly mixed and made into a dough followed by pressing through 2 mm diameter hand extruder. Thereafter, the pellets were dried at room temperature (29 ± 1 °C) for 24 h and stored at 4 °C till use. The inactivated whole cell (IWC) *E. tarda* vaccine was made according to Caipang et al. [30], with minor modifications. An overnight broth culture of *E. tarda* was adjusted to a concentration of 10^6^ CFU/mL in PBS, inactivated for 1 h at 60 °C, and kept at 4 °C until use. The efficiency of inactivation was determined by plating 100 μL of the above bacterial suspension onto tryptic soya agar (TSA, Hi-Media, Mumbai, India) and the sterility monitored for three days. The oral vaccine was prepared at a proportion of 600 μL of IWC–*E. tarda* antigen thoroughly mixed with 120 g commercial feed followed by making a dough and pressing it through a 2 mm diameter hand extruder in the same pattern as preparation of the nanoparticle vaccines.

### 2.6. Vaccination and Challenge Study

**Figure 1:**
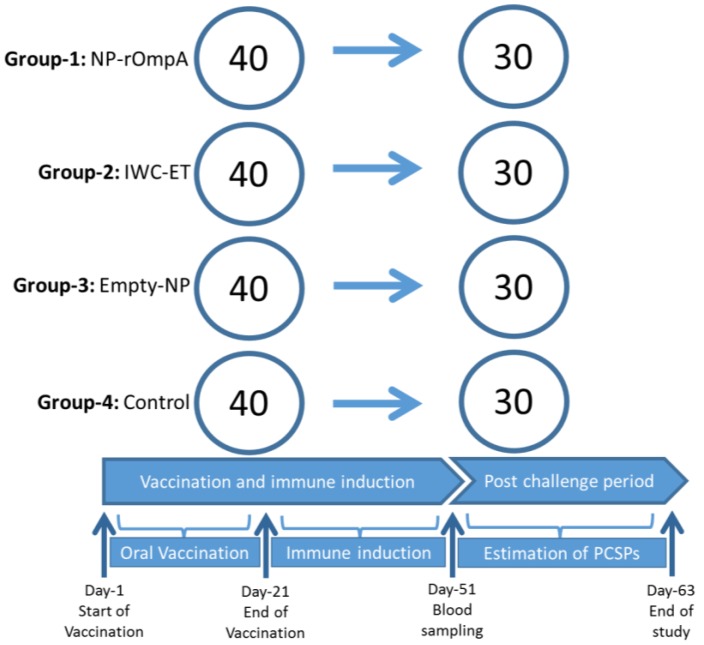
The study design for the oral vaccination trial of carp against *E. tarda* using different vaccines. Four vaccine groups were each allocated 40 fish. Group 1 was allocated the chitosan NP rOmpA vaccine, designated NP-rOmpA. Group 2 was vaccinated with an inactivated whole cell (IWC) *E. tarda* vaccine designated, IWC-ET. Group 3 was vaccinated with empty nanoparticles, without the rOmpA antigen, designated NP-Empty; Group 4 was left unvaccinated and served as the control group. The study timeline for the vaccination trial was segmented into (i) the oral vaccination period of 21 days; (ii) an immune induction period of 51 days post vaccination (dpv); and (iii) the post-challenge period. Blood samples were collected from 10 fish in each group and challenged at 51 dpv. The vaccination trial ended at 63 dpv when fish stopped dying after challenge.

Healthy *L. fimbriatus* weighing approximately 12 g on average were obtained from the Bhadra Reservoir Carp Centre, Karnataka, India and transported to the wet lab of the department in oxygenated bags. The health status of the fish used in this study was determined by collecting a representative random sample of 12 fish that were used for bacteriology, pathology, and histopathology examination. Bacterial pathogens examined were *Aeromonas hydrophila* and *E. tarda*, shown to infect different fish species in India [16,17,31] and the organs examined included the head kidney, spleen, pancreas, liver, gill, heart, and muscle. No bacterial infections, pathology, or histopathological changes were detected in any organs examined from the sampled fish. Fish used for the study were maintained in recirculating water at 28 °C with uniform aeration at a water flow rate of 3.0 L·min^−1^ during acclimatization for a period of one month before performing the immunization experiments. They were fed ad libitum and anesthetized using tricaine methanesulfonate (80 μg/mL) before handling. The 160 fish included in this trial were equally distributed (Figure 1), with 40 in each of the four tanks. Fish in Tank 1 were vaccinated using the NP-rOmpA vaccine while those in Tank 2 were vaccinated with the IWC-ET vaccine. Fish in Tank 3 were vaccinated using empty NPs, designated as NP-Empty without rOmpA antigen, while those in Tank 4 served as an unvaccinated control group. All vaccines were orally administered in feed at 6 μg/g of fish body weight for 21 days. Blood samples were collected from 10 fish from each group at 51 days post-vaccination (dpv), followed by challenge experiment with a pathogenic strain of *E. tarda* (PCF01, 2.4 × 10^8^ CFU/mL) [11] at 51 dpv by intramuscular injection. Mortality was recorded daily until fish stopped dying. Protection was estimated using the Kaplan Meyer’s survival analysis.

### 2.7. Antibody Responses Detected by Enzyme Linked Immunosorbent Assay

Immune response to rOmpA vaccination was determined by measuring the serum antibody titer by ELISA. Briefly, ELISA plates (Greiner Bio-One, Frickenhausen, Germany) were coated with rOmpA (2 μg/well) diluted in carbonate-bicarbonate buffer (pH 9.6) and incubated overnight at 4 °C. Thereafter, the plates were washed in PBS and blocked with 350 μL of 3% BSA at 37 °C for 2 h. After washing, 100 μL fish sera (1:200 dilution) was added to each well and incubated at 37 °C for 2 h. Rabbit anti-rohu HRPO (horseradish peroxidase, DAKO, Sweden) conjugate was added to each well after washing and the plates were incubated at 37 °C for 35 min. The final reaction was obtained by adding tetramethylbenzidine (TMB) hydrogen peroxide substrate to each well and the plates were read at 450 nm absorbance using an EL_X800_ Universal microplate reader (BioTek, Winooski, VT, USA). Serum used in this study was from 10 fish sampled per group that were individually tested in duplicate and the mean OD-value determined was used to compare antibody responses between the vaccinated and control groups.

### 2.8. Serum Inhibitory Assay

The serum-mediated antibacterial activity was measured as described by Hamod et al. [32] with minor modifications. Briefly, 10 μL of overnight grown broth culture of *E. tarda* was adjusted to a density of 10^3^ CFU/mL in PBS, to which 90 μL serum was added in a microtube. The serum plus bacteria suspension was mixed thoroughly, followed by incubation for 24 h at 30 °C. Thereafter, a 10-fold serial dilution of the serum bacteria mix was prepared from each sample and 100 μL of each was spread plated on TSA plates followed by incubation for 24 h at 30 °C. Bacterial colonies were counted and the results expressed as log_10_ CFU/mL. Reduction in bacterial growth was determined by subtracting counts of vaccinated fish from the control group. Serum samples used in this study were from fish from each group sampled at 51 dpv.

### 2.9. Statistical Analysis

All statistical analyses and plotting of graphs were carried out GraphPad version 6. Post-challenge survival analysis were carried out using the Kaplan Meyer’s survival analysis. Differences were considered significant different at *p*-value = 0.05% and confidence limits of 95%.

## 3. Results

### 3.1. Expression, Purification and Concentration of Recombinant OmpA

**Figure 2:**
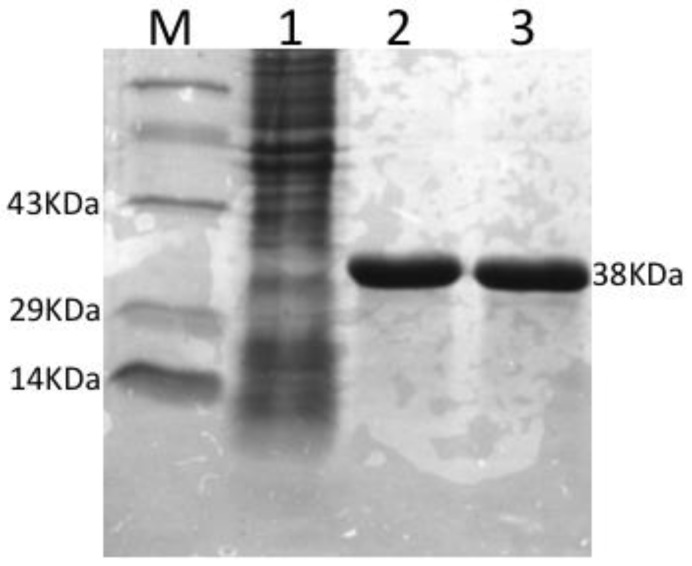
Expression of rOmpA protein. Lane M: Molecular protein marker; Lanes 1: Un-induced recombinant *E. coli* M-15 cell; Lane 2 and 3: Purified rOmpA.

The rOmpA protein was expressed after 4 h induction with 1 mM IPTG. The molecular weight (MW) was estimated to be 38 kDa (Figure 2) by 12% SDS-PAGE with 85% purity and at 1.0 mg/mL concentration.

### 3.2. Physiochemical Properties of Chitosan Nanoparticles Encapsulating rOmpA

**Figure 3:**
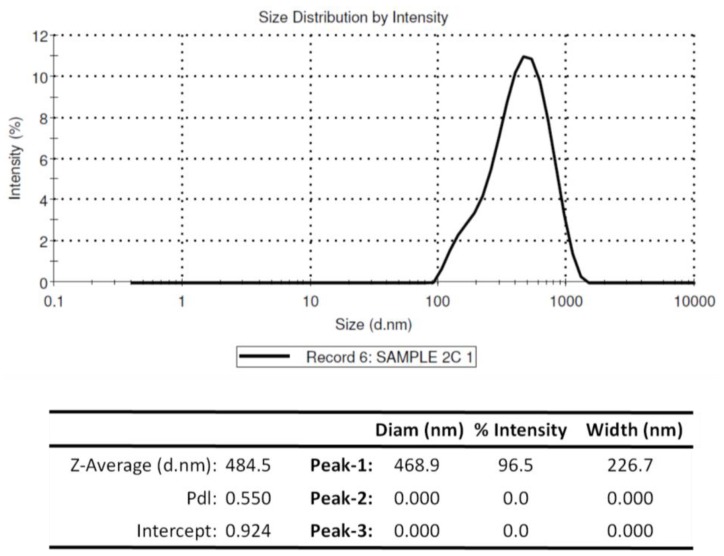
Chitosan nanoparticle size distribution intensity determined using Malvern ZetaSizer (NanoZS; Malvern Instruments, UK; www.malvern.com) at temperature of 25 °C, count rate (kcps) of 407, duration of 60 s and measurement position of 5.50 mm.

**Figure 4:**
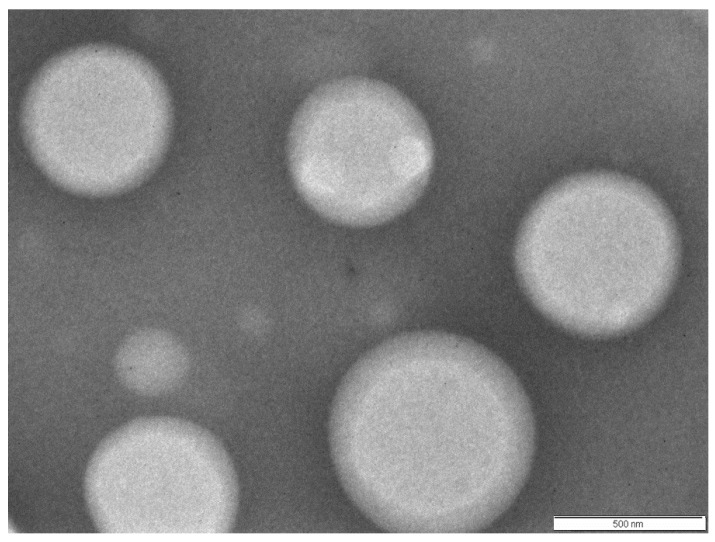
Size and surface morphology of freeze-dried chitosan nanoparticles, as determined by transmission electron microscope (Morgagni TEM, FEI, Eindhoven, The Netherlands).

The encapsulation efficiency of rOmpA antigen by chitosan NPs was estimated to be 60.06%, with the particles having an average diameter of 468.9 nm (Figure 3). Figure 4 shows the size and surface morphology of the freeze-dried chitosan nanoparticles, as determined by transmission electron microscope (Morgagni TEM, FEI, Eindhoven, The Netherlands). The charge of rOmpA encapsulated chitosan NPs was +19.05 mV.

### 3.3. In Vitro Release Assay

**Figure 5:**
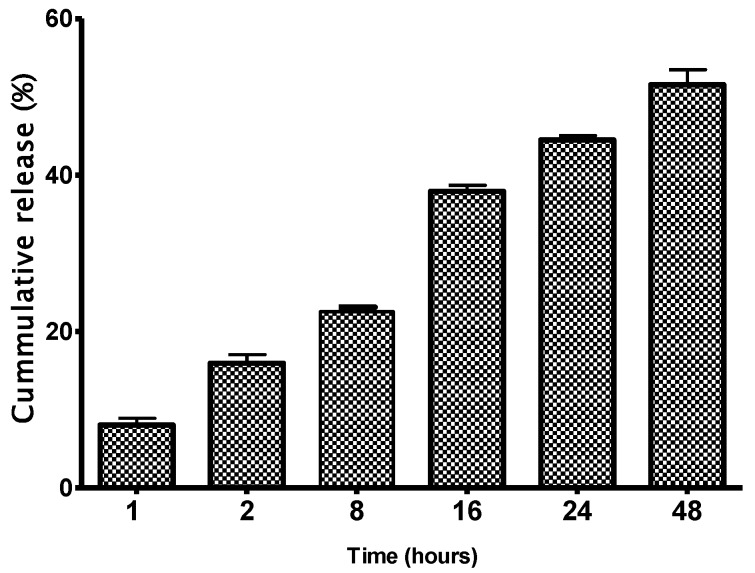
In vitro release of rOmpA protein from encapsulated NPs. Note the exponential increase in rOmpA release within 24 h of the start of the in vitro test.

Figure 5 shows the quantification of rOmpA antigens released from encapsulated chitosan nanoparticles. The recombinant OmpA released from the NPs increased exponentially from less than 10% in 1 h to 49% by 48 h after the start of the in vitro release assay.

### 3.4. Antibody Responses

**Figure 6:**
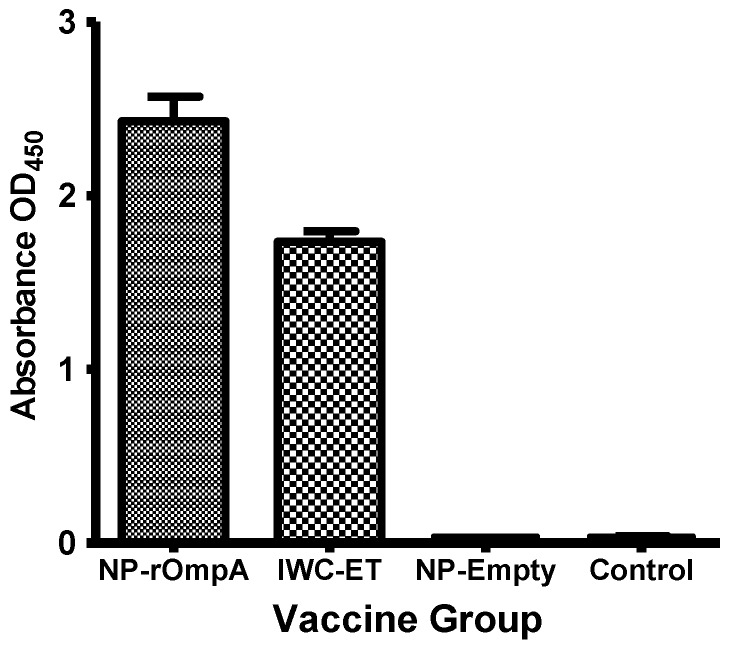
Antibody levels detected against *E. tarda* from fish vaccinated with the NP-rOmpA, IWC-ET, NP-Empty, and control groups at 51 dpv. Antibody levels from fish vaccinated with the NP-rOmpA were higher than antibody levels from those vaccinated with the IWC-ET vaccine. A significant difference (*p* < 0.0003) in antibody levels was detected between the NP-rOmpA and IWC-ET vaccinated groups. The NP-Empty and control groups did not show detectable antibodies against *E. tarda* at 51 dpv.

Circulating antibody levels at 51 dpv in the different vaccine groups are shown in Figure 4. The highest levels were recorded in fish vaccinated with NP-rOmpA vaccine (Mean OD_450_ = 2.430), followed by the IWC-ET (Mean OD_450_ = 1.736). There was a significant difference (*p* = 0.0458) between antibody levels detected in the NP-rOmpA and IWC-ET group. Figure 6 shows that there was no antibody response detected in fish vaccinated using the NP-Empty vaccine and the unvaccinated control group.

### 3.5. Serum Inhibition Test

**Figure 7:**
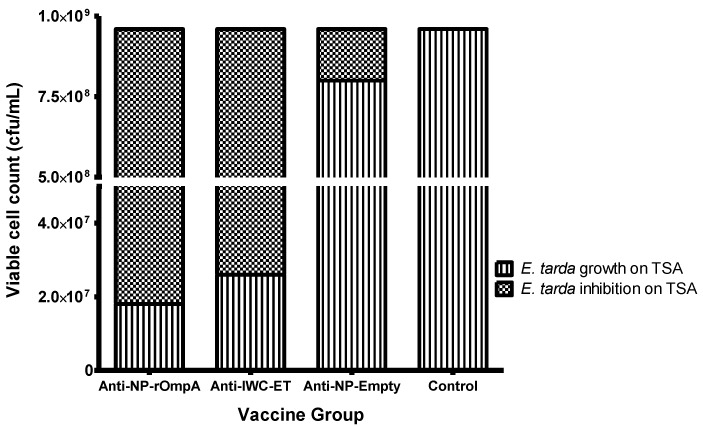
Shows *E. tarda* growth and inhibition on TSA after treatment of sera from vaccinated fish with cultured bacteria. The concentration of the bacteria used was 10^3^ CFU/mL, to which 90 μL serially diluted sera were added in microfuge tubes followed by spread plate technique and incubation on TSA for 24 h at 30 °C. Bacterial growth for each group was determined by counting individual colonies on TSA while inhibition was calculated by subtracting the bacterial colony counts of vaccinated fish from the control group. Note that serum samples from the NP-rOmpA, IWC-ET, and NP-Empty vaccines were designated as anti-NP-rOmpA, anti-IWC-ET, and anti-NP-Empty, respectively. The serum used in this study was pooled from blood samples of 10 fish collected at 51 dpv.

*E. tarda* colony counts obtained on TSA plates after spread plate technique and incubation with a bacteria serum mixture from vaccinated and control fish are shown in Figure 7. Subtracting the colony counts of the vaccinated from control fish shows that the largest inhibition of bacterial growth was from the NP-rOmpA group with a reduction of 10 × 10^7^ CFU/mL, followed by the IWC-ET group, which showed a reduction of 10 × 10^5^ CFU/mL. This shows that fish vaccinated with the NP-rOmpA vaccine had 1.7 times higher inhibitory capacity than the group vaccinated with the IWC-ET vaccine. The group vaccinated with the NP without any antigen added, moderately inhibited *E. tarda* growth compared to the control group but to a lesser extent than the NP-rOmpA and IWC-ET.

### 3.6. Kaplan Meyer’s Survival Analysis

**Figure 8:**
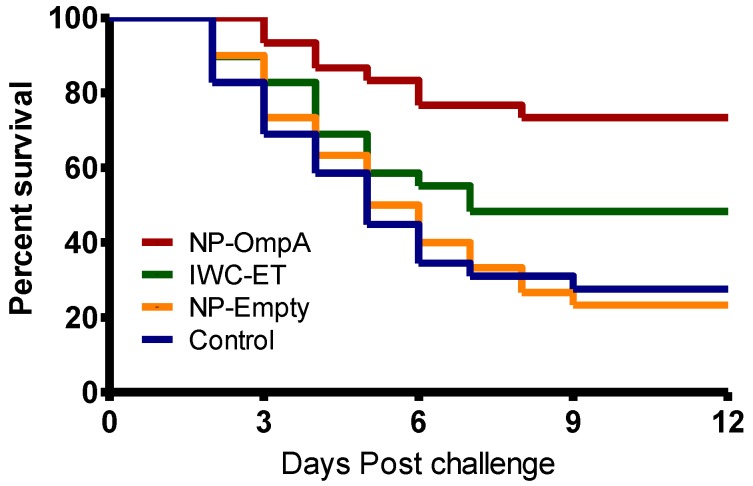
Kaplan Meyer’s survival analysis of fish vaccinated against *E. tarda* using the NP-rOmpA, IWC-ET, and NP-Empty vaccine together with control fish. Mortality in the IWC-ET, NP-Empty, and control groups started two days post challenge (dpc), while in the NP-rOmpA group it started 3 dpc. The highest post-challenge survival proportion (PCSPs) was from the NP-rOmpA group (PCSP = 73.33%), followed by the IWC-ET group (PCSP = 48.28%), while the NP-Empty (PCSP = 23.33%) and control groups (PCSP = 27.59%) had the lowest PCSPs. There was a significant difference (*p* = 0.0396) in PCSP between the NP-rOmpA and IWC-ET groups. No significant difference (*p* = 0.989) was detected in PCSPs between the NP-Empty and control groups.

Figure 8 shows the Kaplan Meyer’s survival analysis of the vaccinated and control groups. Mortality in the IWC-ET, NP-Empty, and control groups started at 2 dpc while in the NP-rOmpA it started at 3 dpc. The highest protection was in the NP-rOmpA group, with post-challenge survival proportions (PCSP) of 73.33%, followed by the IWC-ET (PCSP = 48.28%), NP-Empty (23.33%), and control (PCSP = 27.59) groups. Comparative analysis of the vaccinated groups showed that there was a significant difference (*p* = 0.0396) between the NP-rOmpA and IWC-ET groups. For the NP-Empty group, there was no difference (*p* = 0.989) compared to the unvaccinated control group.

## 4. Discussion

Vaccination is one of the most effective disease control strategies and is the single most important factor for reduction of disease outbreaks in aquaculture [33]. Development of highly efficacious vaccines, particularly for many bacterial infections, is accredited to the use of adjuvants in vaccine formulations that boost the immunogenicity of non-replicative antigens [13] and prolong protection [34]. Koppolu and Zaharoff [35] showed that NPs have the capacity to efficiently deliver encapsulated antigens to activated macrophages and dendritic cells. Moreover, Zaharoff et al. [36] have shown that chitosan NPs enhance humoral and cellular mediated immune responses to vaccination in the absence of adjuvants. In the present study, the efficacy of chitosan NPs as an antigen delivery system for rOmpA and their ability to evoke protective antibody response in carp was evaluated. In our previous study [19], we showed that the infectious pancreatic necrosis virus (IPNV) variable protein 2 (VP2) that had a molecular weight (MW) of 36 KDa was encapsulated in 332 nm PLGA NPs at an encapsulation efficiency of 83%, suggesting that the 468.9 nm diameter chitosan NPs generated in this study were large enough to encapsulate the 38KDa rOmpA protein at a high encapsulation efficiency. However, the 60% encapsulation efficiency obtained in this study is in the range that has been shown to efficiently deliver encapsulated antigens in vivo by chitosan NPs [37,38]. However, at encapsulation efficiency >70% this has been shown to increase drug delivery in vivo [37], implying that the increased encapsulation (of rOmpA) might contribute to even higher protection in vaccinated fish. Given that nanodiameters <500 nm have been shown to be successfully endocytosed by antigen-presenting cells (APCs) [39,40,41], it is likely that the NPs produced in this study had the potential to be endocytosed by APCs in vaccinated fish. Furthermore, the anionic zeta potential of +19.04 mV would enhance uptake by APCs. In the referred study [42], it was surmised that phagocytic cells preferentially take up anionic NPs while cationic NPs are mostly ingested by nonphagocytic cells. It is not known if the same principles apply for fish but delivering NP-rOmpA orally to rohu elicited a systemic immune response (humoral antibodies). To what extent this involves both antigen uptake (over epithelium) and delivery to antigen-presenting cells remains to understood.

The OmpA protein is among the immunogenic proteins expressed on the outer surface of bacterial membranes [43]. Its potential as a vaccine candidate includes its cross-reactivity, surface exposure of antigenic epitopes, and conservation among different strains [43,44,45]. It stimulates macrophages and upregulates MHC-II, CD80, and CD86, expression resulting in activation of CD4^+^ T-cells and an adaptive immune response [45]. It has been shown to induce IgG and IgA both in systemic and mucosal components in higher vertebrates [46] and hence is likely to induce mucosal and systemic immune responses in fish vaccinated by the oral route, using mechanisms similar to those seen in higher vertebrates. In fish, it has been shown to induce protective immunity against *Vibrio harveyi* in Senegalese sole (*Solea senegalensis*, Kaup) [47], *E. tarda* in Japanese flounder (*Paralichthys olivaceus*) [48], *Vibrio anguillarum* in Asian sea bass (*Lates calcarifer*) [49], and *A. hydrophila* in carp [11]. As pointed out by Meenakshi et al. [50], OMPs are only protective in the presence of adjuvants and, hence, it is likely that the higher protection induced by the NP-rOmpA vaccine was due to the adjuvant effect of the chitosan NPs used to deliver the rOmpA antigens in this study [51]. On the contrary, the lower protection levels observed in the IWC-ET group could be due to the lack of an adjuvant that would be able to boost the immunogenicity of IWC antigens and the fact that there was possibly no depot formation for the slow release of the IWC-ET antigen. These factors could account for the low antibody levels detected in the IWC-ET group, resulting in low PCSP in vaccinated fish. On the contrary, the NP-rOmpA group that had an inherent adjuvant effect and a pulsed slow antigen release had high antibody levels that corresponded with high PCSPs in vaccinated fish. Taken together, these findings show that oral vaccination using the OmpA antigens encapsulated in chitosan NP induces higher protection than IWC-ET vaccines without adjuvants in vaccinated fish.

Although different NP vaccines have been used in aquaculture [18,19,52,53,54], few studies have evaluated their ability to release the antigens in vitro [55,56]. It is important that the ability of the NPs to release antigens in vitro be evaluated as this would give insight into their ability to induce a protective immune response in vaccinated fish. In this study, at least 49% of the rOmpA antigen was released within 48 h after onset of the in vitro release test. This observation is in line with that of Ranjan and Nayak [56] who showed an in vitro release of 52%–55.6% of *A. hydrophila* OMPs within 48 h of incubation in PBS. To evaluate the in vivo release of rOmpA, we used the serum inhibition test to evaluate the ability of antibodies induced by rOmpA released in vaccinated fish to inhibit the growth of *E. tarda* on TSA. The serum inhibition test is an in vitro vaccine efficacy measure used to determine the ability of antibodies generated by vaccination to inhibit bacterial growth in vitro [11,57]. Given that in some cases the bacterial strain used for vaccine production is also used as the challenge strain for measuring the RPS of vaccinated fish, in such cases the serum inhibition test serves as an in vitro measure of vaccine efficacy, used to determine the protective ability of antibodies generated by the vaccine strain challenged using its homologous strain [11,57]. As seen from our findings, the NP-rOmpA group had a higher inhibitory capacity than the IWC-ET vaccinated fish, suggesting that the NP-OmpA produced higher levels of protective antibodies than the IWC-ET vaccine. This was supported by ELISA data that showed high antibody levels against *E. tarda*, which corresponded with higher PCSPs for fish vaccinated with the NP-rOmpA vaccine than the IWC-ET vaccine. The importance of serum inhibition tests to demonstrate the in vitro protective ability of antibodies generated by oral vaccination as a measure of in vivo antigen release and vaccine efficacy was found to be significant in this study. However, in order to improve the efficacy of OMPs delivered by NP vaccines, future studies should seek to increase the encapsulation efficiency, elucidate antigen uptake and bio-distribution, and determine the protective mechanism of OMPs and adjuvant effect of NP vaccines, as has been done for other oral vaccines for fish. Nevertheless, in this study we have shown that NP vaccines could serve as an effective oral immunization strategy in fish and would be a better alternative to inactivated whole cell oral vaccines without adjuvants.

## 5. Conclusions

This study has shown that *E. tarda* OmpA encapsulated in chitosan nanoparticles is protective when administered orally in Fringed-Lipped Penisula carp. The study also shows that the protection induced by the OmpA encapsulated in the chitosan nanoparticles was superior to inactivated whole cell vaccine without adjuvants. Hence, there is need to investigated the adjuvant effect of chitosan nanoparticles in fish.
